# A survey of East Palaearctic Lycosidae (Araneae). 7. A new species of *Acantholycosa* Dahl, 1908 from the Russian Far East

**DOI:** 10.3897/zookeys.79.945

**Published:** 2011-02-03

**Authors:** Yuri M. Marusik, Mikhail M. Omelko

**Affiliations:** 1 Institute for Biological Problems of the North, Portovaya Str. 18, Magadan 685000 Russia; 2 Gornotaezhnaya Station FEB RAS, Gornotaezhnoe Vil., Ussuriyski Dist., Primorski krai 692533; 3 Zoological Museum, University of Turku, Turku, FI-20014, Finland

**Keywords:** spiders, East Palaearctic, Asia, new species, key

## Abstract

Acantholycosa azarkinae **sp. n.** is described from the Maritime Province of Russia on the basis of both sexes. Acantholycosa norvegica (Thorell, 1872) is reported from the Maritime Province for the first time. A key and illustrations to all six species that occur in Far East Asia are provided.

## Introduction

Acantholycosa Dahl, 1908 is a relatively small Holarctic genus with 26 species and one subspecies (Platnick 2010). It is a well delimited genus that can easily be recognized by having 4–6 pairs of ventral tibial spines on legs I and II, and a modified palea. The genus was recently revised by Marusik et al. (2004). Acantholycosa has a rather unusual geographical distribution, with two centres of species richness, including an extraordinary degree of endemism in the northern Palaearctic (Marusik et al. 2004). Twenty-one species of this genus are known from the Altai-Sayan mountainous region, of which 17 are local endemics. Four species of Acantholycosa are known from the Maritime Province (Acantholycosa aborigenica Zyuzin & Marusik, 1998; Acantholycosa lignaria (Clerck, 1757), Acantholycosa oligerae Marusik et al., 2004 and Acantholycosa sundukovi Marusik et al., 2004), two of which are local endemics. No other areas in the Holarctic region have more than two species.

While studying wolf spiders in the Maritime Province of Russia we found two additional species, one of which was new to science. The main aim of this paper is to provide a description of the new species. We also review and provide a key to all species known to occur in the whole of the Russian Far East.

## Material and methods

Specimens were photographed using an Olympus Camedia E-520 camera attached to an Olympus SZX16 stereomicroscope in the Zoological Museum, University of Turku. The images were montaged using “CombineZP” image stacking software. Photographs were taken in dishes of different size with paraffin at the bottom. Different sized holes were made in the bottom to keep the specimens in the required position. Figures 6–7, 13–21, 29–40 are reproduced from Marusik et al. (2004) with permission of the coauthors G.N. Azarkina and S. Koponen, in addition to N. Smirnov, the chief editor of Arthropoda Selecta.

The standard of description follows that in Marusik et al. (2004). All measurements are in mm.

The material treated herein will be deposited in the Zoological Museum of the Moscow State University (ZMMU) and in Gornotayozhnaya Station (GTS).

## Species survey

### Acantholycosa azarkinae sp. n.

urn:lsid:zoobank.org:act:8E2A95F1-AC1D-4535-8FB1-2202B4897FF0

Figs 1–5 8–12 26–28 

#### Types.

 Holotype ♂ and paratypes ♀ (ZMMU) and 1♂ 1♀ (GTS) from Russia, Maritime Province, Lazovski District, Sestra Mt., 43°31'52.23"N, 134°02'49.44"E, 1600 m, scree, 16–23.06.2005 (M.M. Omelko).

#### Etymology.

 The specific name is a matronym in honor of our friend and colleague Galina N. Azarkina.

#### Diagnosis.

 The new species can be easily distinguished from other congeners occurring in the Far East by the shape of the palp, which has a broad embolus tip (Figs 8, 10) (not broad in the other species) and by the shape of the epigyne, which has a broad apical pocket and well developed hoods (Figs 26–28).

#### Comments.

 Acantholycosa azarkinae sp. n. is morphologically close to two other endemic species that occur in the Maritime Province: Acantholycosa oligerae and Acantholycosa sundukovi. The three species have similar male palps although they differ from one another by the shape of the tegular apophysis and the embolus.

#### Description

(male(female)). Total length 8.0(8.9). Carapace: 3.7(3.6) long, 3.4(3.1) wide. Carapace and abdomen blackish brown, pattern indistinct. Femora I in both sexes with dark semicircles. Males darker than females. Male leg I with dense black hairs on all segments except for tarsus (Fig. 5). Leg II also with hairs but less dense. Carapace/femur I ratio 1.06(1.0). Leg I segments: 3.5(3.6) + 1.5(1.6) + 3.5(3.5) + 3.5(3.2) + 1.7(1.5). Femur I with 2 dorsal, 2 pro- and 2 retrolateral spines; patella with 1 retrolateral spine (0 in female); tibia I with 1 prolateral and 5 pairs of ventral spines (1p, 1r, 5-5v in females); metatarsus with 1 pro-, 1 retrolateral and 2 pairs of ventral spines.

Male palp as in Figs 1–4, 8–12. Cymbium with 3 claws, tegular apophysis without apical arm, palea with laminar outgrowth, terminal apophysis large with claw-like tip; embolic base with small, almost indistinct “spine”, tip of embolus widened and subdivided into two lobes.

Epigyne as in Figs 26–28. Apical pocket wide with two distinct hoods, septum distinct, septum with trapezoidal base; spermathecae long, with blind outgrowth in basal third.

**Figures 1–7.**
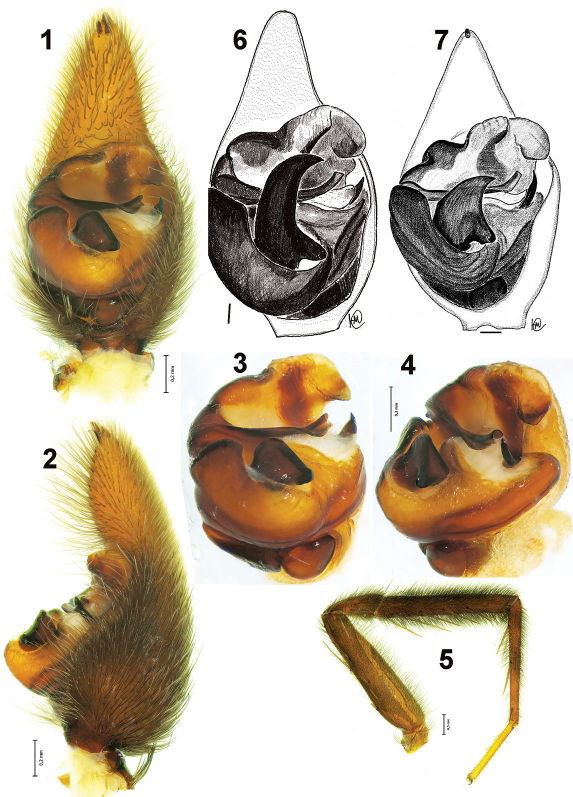
Male palp and leg I of Acantholycosa azarkinae sp. n. (**1–5**), Acantholycosa oligerae (**6**) and Acantholycosa sundukovi (**7**). **1, 6, 7** male palp, ventral **2** male palp, retrolateral **3** bulbus, ventral **4** bulbus, retrolateral **5** leg I, prolateral. **6–7** after Marusik et al. (2004). Scale = 0.1 if not otherwise indicated.

**Figures 8–21.**
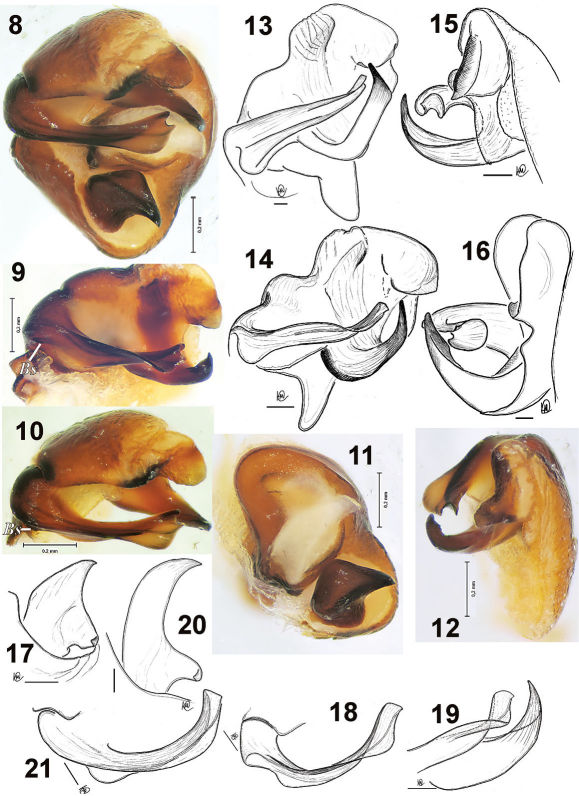
Male palp of Acantholycosa azarkinae sp. n. (**8–12**), Acantholycosa oligerae (**13–14, 20–21**) and Acantholycosa sundukovi (**15–16, 17–19**). **8** bulbus, from above **9, 13, 15** terminal part of palp, ventral **10** terminal part of palp, from above **11** tegulum, from above **12, 14, 16** terminal part of palp, retrolateral **17, 20** tegular apophysis, ventral **18, 21** embolus, from above **19** terminal part of embolus and terminal apophysis. **13–21** after Marusik et al. (2004). Scale = 0.1 mm if not otherwise indicated.Abbreviations: *Bs =* basal spine of embolus.

**Figures 22–25.**
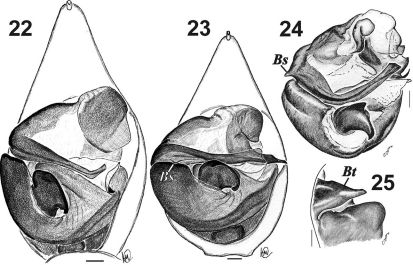
Male palp of Acantholycosa aborigenica (**22**), Acantholycosa lignaria (**23**) and Acantholycosa norvegica (**24–25**). **22–23** ventral **24** bulbus, ventral **25** base of embolus showing tooth. All after Marusik et al. (2004). Scale = 0.1 mm.Abbreviations: *Bs =* basal spine of embolus.

#### Distribution.

 Type locality only.

### Acantholycosa aborigenica

Zyuzin & Marusik, 1988

Figs 22 35–36

Acantholycosa aborigenica Zyuzin and Marusik 1988: 1083, f. 1–6 (♂♀).
Acantholycosa aborigenica : Marusik et al. 2004: 123, f. 108–114, 125–127, 147–151 (♂♀).

#### Material examined.

 4♂ 3♀ (GTS), Russia, Maritime Province, Ussuriyski District, environs of Gornotaezhnoe Village, Kamenistaya Sopka, 43°42'22.02"N, 132°07'30.93"E, 218 m, stones, 19–26.06.2010 (M.M. Omelko); 3♂ (GTS), same locality, 02.07.2010 (M.M. Omelko).

#### Comments.

 This species has been well described in the two publications mentioned above. It is distributed from Central Aimak in Mongolia to Kolyma River, and south to Maritime Province (Marusik et al. 2004). Within the Far East it has been reported from the upper Kolyma, northern Cisokhotia, as well as from the Khasan and Ussuriyski districts of Maritime Province.

**Figures 26–34.**
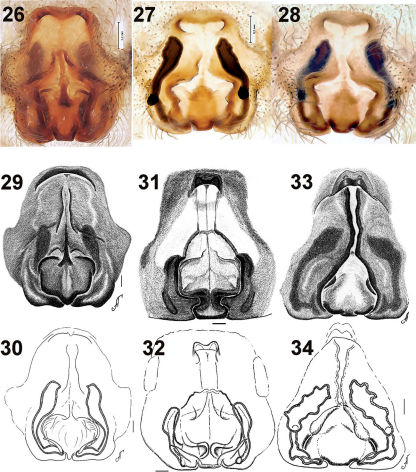
Epigyne of Acantholycosa azarkinae sp. n. (**26–28**), Acantholycosa oligerae (**29–30**), Acantholycosa norvegica (**31–32**) and Acantholycosa lignaria (**33–34**). **26, 28–29, 31, 33** epigyne, ventral **27, 30, 32, 34** vulva, dorsal. **27–28, 30, 32, 34** after maceration. **29–34** after Marusik et al. (2004). Scale = 0.1 mm if not otherwise indicated.

### Acantholycosa lignaria (Clerck, 1757)

Figs 23 33–34 37

Acantholycosa lignaria : Holm 1947: 37, pl. 8, f. 82–83, pl. 10, f. 47 (♂♀).
Acantholycosa lignaria : Marusik et al. 2004: 119, f. 27–29, 54, 115–121 (♂♀).

#### Material examined.

 2♀ (GTS), Russia, Maritime Province, Chuguevskii District, Oblachnaya Mt., 43°41'43.75"N, 134°12'00.04"E, 600 m, fallen tree-trunks, 11–15.08.2003 (M.M. Omelko).

#### Comments.

 This species has been well described in several publications. It has a trans-Palaearctic range (Marusik et al. 2004). Previously it was reported from Ussuri Reserve (Marusik et al. 2004). Unlike other Acantholycosa species this species lives in habitats without stones. From other congeners it can be easily distinguished by having only 4 pairs of ventral tibial spines (other species have 5–6 pairs).

**Figures 35–40.**
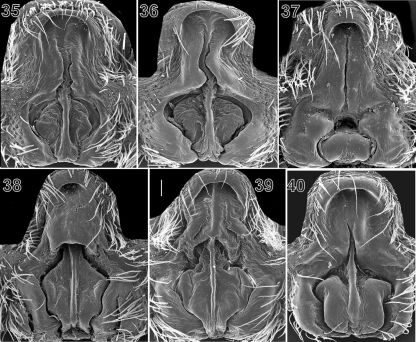
SEM microphotographs of epigyne of Acantholycosa aborigenica (**35–36**), Acantholycosa lignaria (**37**), Acantholycosa norvegica (**38–39**) and Acantholycosa oligerae (**40**). All after Marusik et al. (2004). Scale = 0.1 mm.

### Acantholycosa norvegica

(Thorell, 1872)

Figs 24–25 31–32 38–39

Acantholycosa norvegica : Holm 1947: 38, f. 4a, 15–16, pl. 8, f. 84–85 (♂♀).
Acantholycosa norvegica : Marusik et al. 2004: 128, f. 92–97, 122–124, 168–172, 181–182 (♂♀).

#### Material examined.

 5♂ 3♀ (GTS), Russia, Maritime Province, Chuguevskii District, Oblachnaya Mt., 43°41'43.75"N, 134°12'00.04"E, 1750 m, high mountain birch wood, 23.06.2008, (M.M. Omelko); 39♂ 4♀ (GTS), same locality, bush thicket, 23.06.2008 (M.M. Omelko).

#### Comments.

 Acantholycosa norvegica is the type species of the genus. It has been well described in several publications. This species has a trans-Palaearctic range. Although it has a wide range and is known from the adjacent Khabarovsk Province (Trilikauskas 2007) and the more eastern Magadan Area, it has not previously been reported from the Maritime Province. It is worth mentioning that the record from Maritime Province is the southernmost of its known range.

### Acantholycosa oligerae

Marusik, Azarkina & Koponen, 2004

Figs 13–14 20–21 26 29–30 40

Acantholycosa oligerae Marusik et al. 2004: 126, f. 19–20, 128, 152–161 (♂♀).

#### Comments.

 This species was recently described from material found at a single locality in the Lazo Reserve, the Russian Far East.

### Acantholycosa sundukovi

Marusik, Azarkina & Koponen, 2004

Figs 15–16, 17–19

Acantholycosa sundukovi Marusik et al. 2004: 128, f. 162–167 (♂).

#### Comments.

 This species is known from the holotype male only. So far, Acantholycosa sundukovi is known from a single locality in the Lazo Reserve (Kordon Amerika), the Russian Far East.

## Key to the Far Eastern Acantholycosa

**Table:** 

1	Males	2
–	Females (♀ of Acantholycosa sundukovi – unknown)	7
2	Embolus with large basal spine (Fig. 25); palea with non laminar outgrowth (Fig. 24), terminal apophysis with fine spine (Fig. 24)	Acantholycosa norvegica
–	Embolus without basal spine or spine is small, almost indistinct; palea with laminar outgrowth	3
3	Tegular apophysis longer than wide due to well developed apical arm (Figs 6–7)	4
–	Tegular apophysis wider than long, apical arm absent or small	5
4	Apical arm of tegular apophysis 1.5 times longer than width of apophysis (Fig. 6); tibia I with 6 pairs of ventral spines	Acantholycosa oligerae
–	Apical arm of tegular apophysis as long as width of apophysis (Fig. 7); tibia I with 5 pairs of ventral spines	Acantholycosa sundukovi
5	Tibia-metatarsus I and II with long hairs (Fig. 5); tip of embolus broad and twisted (Figs 1, 3–4, 8–12)	Acantholycosa azarkinae sp.n.
–	Legs I and II without long hairs; tip of embolus not broad and not twisted	6
6	Tip of embolus bent (Fig. 22); paleal outgrowth larger than tegular apophysis; tibia I with 5–6 pairs of ventral spines	Acantholycosa aborigenica
–	Tip of embolus not bent (Fig. 23); paleal outgrowth smaller than tegular apophysis; tibia I with 4 pairs of ventral spines	Acantholycosa lignaria
7	Apical pocket thinner than septal width (Figs 31, 33, 37–39)	8
–	Apical pocket wider or subequal to width of septum (Figs 26–28, 35–36)	9
8	Fovea and septum triangle-shaped (Figs 33, 37), stem of septum lies in thin furrow; tibia I with 4 pairs of ventral spines	Acantholycosa lignaria
–	Fovea and septum square or round in shape (Figs 31–32, 38–39); tibia I with 5 pairs of ventral spines	Acantholycosa norvegica
9	Metatarsus I with 3 pairs of ventral spines; femur I with one retrolateral spine	Acantholycosa oligerae
–	Metatarsus I with 2 pairs of ventral spines; femur I with 2 retrolateral spines	10
10	Fovea with rounded sides, apical pocket undivided (Figs 35–36)	Acantholycosa aborigenica
–	Fovea with straight sides (Figs 26–28), apical pocket with two distinct hoods	Acantholycosa azarkinae sp.n.

## Conclusions

The number of Acantholycosa species in the Maritime Province of Russia is fewer than that of the Altai-Sayan region only, with 6 and 21 species respectively. The same is true for the number of endemic species (3 and 17 respectively). Presently, only the southern region of the Maritime Province has been relatively well studied. The huge territories of Sikhote-Alin remain uninvestigated. Given the high level of endemism among petrophilous species of spiders it is reasonable to expect the occurrence of additional new species in the province, especially on isolated screes on mountain tops.

## Supplementary Material

XML Treatment for Acantholycosa azarkinae

XML Treatment for Acantholycosa aborigenica

XML Treatment for Acantholycosa lignaria

XML Treatment for Acantholycosa norvegica

XML Treatment for Acantholycosa oligerae

XML Treatment for Acantholycosa sundukovi
